# Emerging Nano-Carrier Strategies for Brain Tumor Drug Delivery and Considerations for Clinical Translation

**DOI:** 10.3390/pharmaceutics13081193

**Published:** 2021-08-03

**Authors:** David J. Lundy, Helen Nguyễn, Patrick C. H. Hsieh

**Affiliations:** 1 Graduate Institute of Biomedical Materials & Tissue Engineering, Taipei Medical University, Taipei 110, Taiwan; m825109009@tmu.edu.tw; 2 International PhD Program in Biomedical Engineering, Taipei Medical University, Taipei 110, Taiwan; 3 Institute of Biomedical Sciences, Academia Sinica, Taipei 115, Taiwan

**Keywords:** blood–brain barrier, nanomedicine, liposome, brain tumor, exosome, glioblastoma multiforme

## Abstract

Treatment of brain tumors is challenging since the blood–brain tumor barrier prevents chemotherapy drugs from reaching the tumor site in sufficient concentrations. Nanomedicines have great potential for therapy of brain disorders but are still uncommon in clinical use despite decades of research and development. Here, we provide an update on nano-carrier strategies for improving brain drug delivery for treatment of brain tumors, focusing on liposomes, extracellular vesicles and biomimetic strategies as the most clinically feasible strategies. Finally, we describe the obstacles in translation of these technologies including pre-clinical models, analytical methods and regulatory issues.

## 1. Introduction

### 1.1. The Blood–Brain Barrier

The blood–brain barrier (BBB) is a complex semi-permeable interface which separates the brain parenchyma from systemic blood circulation at the microvascular level. The barrier is formed from interlinked brain endothelial cells (BECs) supported by pericytes and astrocyte end feet, which cover the majority of BEC surface area. Together with neurons these cells form the neurovascular unit (NVU). Pericytes alter BEC gene expression, and astrocytes release growth factors capable of up- or down-regulating barrier function, particularly in response to injury [1,2].

The human brain requires a disproportionately large amount of oxygen and glucose, and thus receives a large amount of cardiac output. With no means to store glycogen, respiratory substrates must be constantly delivered “on demand”. To accomplish this, the average adult human brain contains around 550 km of brain capillaries and a total surface area of approximately 12 m^2^ for nutrient exchange [3]. The BBB preserves brain homeostasis by protecting the sensitive brain from toxins, blood cells and pathogens which may enter from the blood.

To nanomedicine researchers interested in brain drug delivery, this 12 m^2^ surface is their target. This review focuses on the barrier properties and methods for bypassing, penetrating or disrupting the BBB for drug delivery. However, it is important to remember that disruption of the BBB is also implicated in the pathogenesis of multiple neurological disorders. Thus, targeting the BBB for therapeutic restoration of function is also clinically important, and any disruption would ideally be minimal and temporary [4,5]. The BBB, despite the name, is also not only a barrier and is perhaps more accurately described as an “interface” possessing multiple functions. For example, the BBB is a target for various hormones, functions as a secretory body, and controls passage of hormones in and out of the brain—thus it may also be considered as an endocrine tissue [6].

The barrier is highly specialized and highly regulated to control the passage of substances between the luminal (blood-facing) side and the abluminal (brain-facing) side. BECs themselves are highly specialized, lacking fenestrations and having very low rates of transcytosis [7]. Furthermore, BECs form tight junctions utilizing occludin, claudin-5 and zonula occludens-1 (ZO-1) to prevent paracellular movement of water-soluble compounds [8]. Therefore, water-soluble substances require specific carriers or transporters to cross the BBB. As such, a wide array of transporters are present to control the influx or efflux of ions, minerals, energy substrates, amino acids, metabolites and proteins [4]. As will be discussed later, these transporters may be “hijacked” to carry desired molecules into the brain via conjugation of natural or artificial ligands onto nanomedicines. To quantify the barrier function, electrical resistance is used to measure transcellular passage of ions. The in vivo BBB is estimated to have an electrical resistance of 1500–2000 Ohms cm^2^, compared to peripheral endothelial barriers which have a resistance of only 3–33 Ohms per cm^2^ [9].

In addition to physical barriers of paracellular and transcellular movement, there is also a so-called “metabolic barrier” where enzymes are able to rapidly alter unwanted metabolites, peptides, hormones and other products which crossed into the brain parenchyma [10]. Finally, a diverse array of multi-substrate efflux pumps including ABCB1/MDR1 (p-glycoprotein), ABCC1 (MRP1) and BCRP/ABCG2, actively transport unwanted substances back to the luminal side, including most drugs of potential value for neurological disorders [11]. These transporters are widely expressed, not only on BECs, but also on astrocytes, neurons, microglia and pericytes. Luminal and abluminal sides have specific expression patterns, which vary by brain locale, forming a heterogenous interface for molecule passage [12]. Adding to this complexity, there is considerable BBB heterogeneity throughout different brain regions, variation by routes of administration, and variations due to disease state [7,13].

### 1.2. The BBB as an Obstacle for Brain Tumor Drug Delivery

In terms of molecule permeability, some general rules for blood-to-brain drug transport have been established, although they are not without exceptions [14]. Molecules larger than 400 Da are very unlikely to cross the BBB, especially if highly water soluble, unless a suitable specific transporter is present. On the other hand, lipid-soluble small molecules tend to have better permeability. For example, the DNA alkylating drug Temozolomide (TMZ), with a molecular weight of 194.154 g/mol and poor solubility in water, readily crosses the BBB and is one of the few treatments available for glioblastoma multiforme (GBM) [15,16]. However, it should be noted that a high degree of lipid solubility alone does not guarantee successful accumulation of meaningful drug concentrations in the brain, since drugs may still be metabolized or removed by efflux pumps [17,18]. Drugs bound to plasma proteins are also unavailable for crossing the BBB, since those proteins may only cross the BBB via specific transporters. This can be demonstrated using an albumin-binding dye called Evans blue which is a standard model used to measure BBB permeability. Albumin is almost completely unable to cross the intact BBB [19]. In summation, these restrictions, along with Lipinski “rule of five” characteristics, mean that most currently used chemotherapeutics are unamenable to brain delivery without methods to improve their BBB passage. Alternatively, new molecules can be designed to avoid efflux pump binding, while meeting above-mentioned criteria of solubility and molecular weight.

### 1.3. Brain Tumors and Current Therapies

This review will focus primarily on nanocarrier-based treatments for glioblastoma multiforme (GBM), which is the most common primary brain cancer. GBM is an aggressive, highly infiltrative disease originating from glial cells, with dire survival rates and few treatment options. Current treatment of GBM relies on surgical resection of the tumor mass, if possible, followed by chemotherapy and radiation [20]. For chemotherapy, the oral DNA alkylating drug Temozolomide is the first drug of choice. TMZ is one of the few chemotherapeutics which passes readily through the BBB. Unfortunately, tumor drug resistance is common, accomplished by increasing methylguanine-DNA-methyltransferase (MGMT) expression by demethylating its promoter site. Following surgical resection, biodegradable wafers (Gliadel^®^ implants) can be implanted which provide controlled release of Carmustine which provide a small benefit (1.1–3.3 months) in overall survival [21]. The humanized anti-VEGF monoclonal antibody Bevacizumab is used for recurrent GBM and provides symptomatic improvement, mainly by reduction in edema due to vascular normalization [18]. Finally, tumor-treating fields (TTFs) are a new strategy which uses electrodes placed on the skin to deliver low intensity electrical fields which disrupt GBM cell mitosis. In a human Phase III clinical trial of 695 GBM patients (NCT00916409), the addition of TTF to TMZ therapy increased overall survival from a median of 16.0 months to 20.9 months [22].

### 1.4. Avoidance, Bypass and Disruption of the BBB

Some non-nanomedicine methods have been used clinically for improving drug delivery to brain tumors. The US FDA approves the use of arterial mannitol infusions for enhancing delivery of chemotherapeutic agents to brain tumors. Hyperosmotic solutions cause rapid shrinkage of BECs, temporarily increasing the rate of transcytosis and disrupting TJs [23]. In addition, the use of transcranial focused ultrasound is a highly promising, less-invasive method for improving drug delivery to targeted brain regions, including tumors [24,25,26]. An excellent summary of clinical trials of BBB disruption is presented elsewhere [27]. It is also possible to bypass some aspects of the BBB altogether by alternative routes of administration. For example, intrathecal chemotherapy, administering drugs directly into cerebrospinal fluid (CSF) by lumbar puncture or intraventricular injection has been used, but not all drugs are amenable to this delivery method [28]. Intranasal administration is being actively explored in pre-clinical trials for brain drug delivery, but this presents new challenges such as drug clearance, degradation of some therapeutics, and the very limited volumes that can be delivered by this route [29]. Implantation of degradable Carmustine-loaded Gliadel^®^ wafers into spaces remaining after tumor resection has been shown to produce slight improvements in survival, albeit with side effects such as edema, risk of infection and interference with subsequent MRI [21]. Convection-enhanced delivery (CED) using an implanted pump to deliver chemotherapy agents directly to tumors has also been used. While this does successfully deliver a high concentration of drugs, it is invasive and carries risk of infection and toxicity [30].

Still, with current treatment regimes, overall survival for GBM is extremely poor and new therapies are desperately needed. Nanomedicines are one approach by which otherwise unsuitable molecules may be carried into the brain by conjugating them or encapsulating them with suitable carriers.

## 2. Nanomedicines

### Nanomedicines as Carriers for BBB Passage

**Figure 1:**
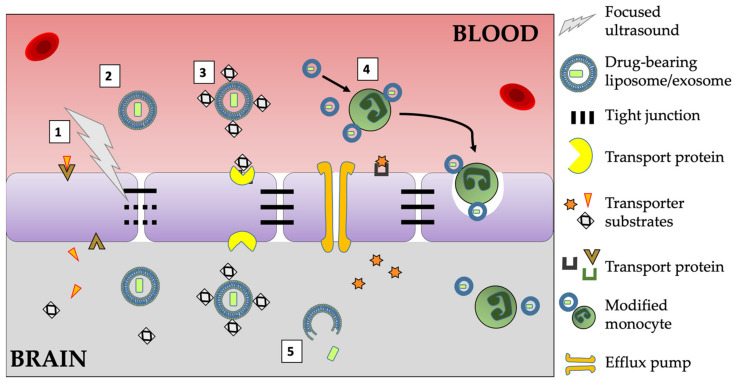
Common routes of nanomedicines targeting brain tumor drug delivery. A schematic diagram (not to scale) shows four main routes by which nanocarriers improve drug delivery to the brain parenchyma. 1. Focused ultrasound weakens endothelial tight junctions and improves uptake of nanocarriers. 2. Liposome passage through the intact BBB occurs, but only at low rates. Exosomes show higher penetration. Both may be enhanced by leaky tumor vasculature known as the enhanced permeability and retention (EPR) effect. 3. Liposomes can be modified with transporter substrates such as glucose, transferrin or glutathione to improve BBB crossing. 4. Liposomes targeting circulating cells capable of BBB penetration, known as the “Trojan Horse” approach. 5. Drugs must eventually leave the liposome in order to act on their targets. However, efflux pumps are capable of removing many drugs from the brain parenchyma.

Nanomedicines are broadly defined as materials with individual diameters of 1–100 nm which have therapeutic or diagnostic applications [31]. The maximal cut-off of 100 nm is somewhat arbitrary, since there is no specific biological or chemical change which occurs once particles exceed this size. Nanomedicine formulations are an extremely attractive proposition for BBB drug delivery, since they can be used to carry molecules which are otherwise BBB impermeable. Their nanoscale size means that they are able to travel through, and penetrate, small capillaries, and their physicochemical properties can be controlled to alter their biodistribution [32]. A schematic diagram showing four main routes of nanocarrier drug delivery across the BBB is shown in Figure 1.

The physiochemical properties of nanomedicines are key determinants of their behavior inside the body [32,33]. Diameter, shape (spherical, rod-shape), surface area (rough, smooth), surface charge and surface chemistry are all important for dictating their pharmacokinetic and pharmacodynamic properties, including preferential accumulation in target tissues such as the brain [34,35]. A recent study by Brown and colleagues comprehensively investigated the effect of size, shape, composition and stiffness of nanoparticle passage through using a human BBB model, combining inhibitors of clathrin and caveolin-mediated endocytosis to tease apart the effects of each variable on the mechanism of uptake. Although there was a size-dependent effect, they found that particle composition was the most important determinant, even more so than size. For example, 500 nm transferrin nanoparticles crossed the barrier more readily than polystyrene nanoparticles or liposomes of the same, or smaller, sizes [36].

It is also well-described that disease states alter both BBB function and distribution of nanomedicines in the body [33,37,38,39]. Houston and colleagues carried out an extremely important study, using mice with spontaneous brain tumor generation, to investigate the relationship between blood–brain tumor barrier (BBTB) permeability to nanomedicines and tumor progression [40]. They found that tumor volume alone was a poor predictor of BBTB permeability compared to changes in post-contrast MRI scans which indicate leakiness. Smaller nanocarriers (20 nm) were able to accumulate in the early stages of tumor development, whereas 100 nm nanocarriers could not cross the BBTB until later stages, which correlated with greater tumor leakiness. In addition, they also found that BBTB permeability varied by brain location, demonstrating that an individualized approach is optimal. This heterogeneity is a challenge in the field, which will be discussed later.

Here, we review the nano-carrier systems which we believe are most suitable for clinical translation: liposomes and polymeric micelles, exosomes, and some biomimetic systems. Other nanomedicine formulations for BBB drug delivery, including metal particles and silica are reviewed elsewhere [41]. A selection of key studies are highlighted in Table 1. These include first-in-human trials of nanomedicine formulations and basic research which is noteworthy due to novelty or a display of outstanding efficacy in animal models. Many of these studies will be discussed in the following sections.

## 3. Liposomes as Nanocarriers for BBB Drug Delivery

Liposomes are the most developed nanoscale drug delivery vehicle and have already seen clinical use in multiple applications from chemotherapeutics to antibiotics, analgesics and vaccines [63]. Liposomes are spherical vesicles formed from natural or synthetic lipids with an inner aqueous center. The lipid composition determines properties such as rigidity, phase transition temperature and stability, which affect drug encapsulation, retention and release [64]. Molecules may be packaged into the aqueous center or, in the case of lipid-soluble drugs, into the hydrophobic portion of the bilayer membrane itself. A balance must be found between liposome stability which prolongs circulatory time and protects the drug from excretion, versus eventual drug release which is essential for its function. Liposome outer membrane lipids often conjugate polyethylene glycol (PEG) which forms a shield around the liposome, protecting it from the reticuloendothelial system, reducing immunogenicity and extending circulation time [63]. A dense PEG coating also improves penetration through brain tissues [65]. However, it is worth noting that although PEG is generally considered as safe, some individuals form anti-PEG IgG and IgM antibodies and complement activation which reduces drug efficacy [66]. In some individuals, severe reactions may occur [67]. For clinical translation, liposomes generally improve the safety and efficacy of the encapsulated drugs by extending circulation time and reducing their off-target accumulation. Several commercial liposome products show that liposomes can be manufactured on large scales, and their synthetic nature makes it easier to assure their batch-to-batch consistency [68]. An elegant paper from Nance et al. calculated that a 100 nm nanoparticle would ideally contain approximately nine PEG molecules (5 kDa) per 100 nm^2^ of particle surface area for optimal passage through brain parenchyma [65].

### 3.1. Passive Liposome Uptake by Brain Tumors

For BBB targeting, liposomes are a very attractive option, particularly for treatment of brain tumors. This is demonstrated by a study by Gao and colleagues who encapsulated Temozolomide (TMZ) into simple phospholipid/cholesterol 156.7 ± 11.4 nm liposomes, which improved pharmacokinetic properties and brain uptake compared to the free drug [69]. This is encouraging, given that TMZ already has excellent brain uptake as a free drug.

Liposomes have three main routes by which they may deliver their cargo into the brain: Transporter-mediated transcytosis (TMT), adsorptive-mediated transcytosis (AMT) and receptor-mediated transcytosis (RMT). AMT is a non-specific process, whereas TMT and RMT occur via reactions with specific brain endothelial cell proteins such as the transferrin receptor (TfR) or GSH glutathione transporter [68]. A fourth route, cell-mediated transcytosis (CMT), often named “trojan horse method” will be described later. These routes are illustrated in Figure 1.

By default, PEGylated liposomes have very poor uptake by healthy brain and by tumors. In a recent study, our group found less than 0.5% of infused intravenous PEGylated liposomal doxorubicin was able to cross the intact BBB and enter the brain of pigs. In mice, this was less than 0.1% of the injected dose [59]. Notably, this is still far greater than free Doxorubicin which was only 0.011% in our study and 0.02% in another published work [47]. Many tumors are known to have chaotic, leaky vasculature compared to normal tissues, resulting in an enhanced permeability and retention (EPR) effect [70]. However, tumors within the brain can still be well protected by the blood–brain–tumor barrier (BBTB) [71]. In mice, we found that an orthotopic xenograft human GBM tumor showed only a 2.3-fold increase in LipoDox accumulation compared to normal brain tissue, representing a very small EPR effect. For comparison, the same GBM tumor implanted subcutaneously accumulated 25.8 times more LipoDox, clearly demonstrating that the BBTB protects tumors from systemically administered liposomes [59]. In addition, the EPR effect is heterogenous between individuals, between brain areas, and even between specific regions of tumors. A clinical trial examining the addition of PEGylated liposomal doxorubicin to a TMZ treatment regime found no benefit in treating GBM patients [42].

Thus, it is clear that liposomes require some “help” beyond passive uptake in order to sufficiently enter the brain [72]. This can be accomplished by pre-weakening of the BBB, or by modifying the liposome itself with targeting, penetrating or shielding properties. As an example of the former, our group has previously shown that use of vascular endothelial growth factor (VEGF) to temporarily open the BBB is an effective method to improve PEGylated liposome drug delivery to brain tumors [59]. We also demonstrated that the same technique is applicable to small molecules, nanoparticles and antibodies.

Since drugs encapsulated inside liposomes are not therapeutically available, one of the popular concepts in the field is using temperature, pH, or other means to trigger drug release from liposomes. Bredlau et al. recently carried out a canine study combining technologies using intravenous thermo-sensitive Doxorubicin-loaded liposomes and local hyperthermia induced by a probe inserted directly into the brain [55]. Although they did not use a tumor model, they confirmed therapeutically relevant Doxorubicin concentrations in brain tissue surrounding the probe as a proof of concept. However, there was localized brain damage and some of the animals displayed side effects. This approach has been investigated for more than 20 years to overcome limitations in liposomal drug release, but it has not yet seen clinical use [73,74,75].

Non-targeted liposomes have been explored clinically for brain drug delivery. Non-PEGylated liposomal doxorubicin (Myocet) was used in 13 children with recurrent glioma (NCT02861222) and liposomal Irinotecan is also being explored for recurrent high-grade glioma (NCT02022644) [52]. Notably, free drug Irinotecan had previously failed a Phase II trial in brain tumor patients [76]. A nanoliposomal form of Irinotecan (NL CPT-11) was also investigated (NCT00734682) in a Phase I study starting in 2008. However, the trial has not been updated since 2015 and we are unable to find published results from the study. Another liposomal Irinotecan (MM-398) recently concluded a Phase I trial in breast cancer with brain metastasis (NCT01770353) and showed some anti-tumor activity in heavily pretreated patients [60].

In our view, passive uptake of liposomes alone is unlikely to become a game-changer for brain tumor treatment. However, focused transcranial ultrasound (FUS) and intravenous microbubbles, mentioned previously, may also be combined with nanomedicine therapy, pre-emptively disrupting the BBB prior to delivery of liposomal chemotherapeutics to brain tumors [51,77]. Ultrasound has been known to modulate the BBB since the 1950’s [78]. Decades of research and development have presented numerous challenges including scale-up to human skull thickness, efficacy and safety concerns over BBB damage and sterile inflammation [79]. Small-scale human trials are now underway. A recent small human trial of four patients conducted by Kullervo Hynynen’s group showed the feasibility of combining FUS with liposomal Doxorubicin or Temozolomide. They were able to collect non-sonicated tumor margin tissue from a single patient and found a 7.7-fold increase in drug concentration in the FUS-treated region [24].

### 3.2. Liposomes Engineered for Brain Tumor Targeting

Rather than relying only on the EPR effect, liposome formulations with integrated targeting are one strategy which is being actively explored. Liposomes may be targeted to the BBB or to aspects of the GBM tumor. Here, we highlight some studies with an emphasis on clinical translation. Gaillard and colleagues modified a commercial ~95 nm PEGylated liposomal doxorubicin with glutathione, targeting the GSH transporter, to improve BBB penetration, which was termed 2B3-101 (since re-named to 2X-111). They showed a roughly two-fold increase in delivery compared to non-modified LipoDox and around ten-fold compared to the free drug, resulting in improved tumor clearance in a mouse model [47]. In a rodent study using cerebral open flow microperfusion, this same formulation showed a 4.8-fold higher doxorubicin dose in the brain compared to non-modified liposome (Caelyx^®^) [48]. In a Phase I/II trial (NCT01386580) 2B3-101/2X-111 was administered to 28 patients with brain cancer and showed a good safety profile and preliminary evidence of efficacy [49]. However, the trial has not been updated since 2015 and we are unable to find any recent 2B3-101/2X-111 publications.

C225-ILs-Dox is a doxorubicin-loaded, PEGylated immunoliposome with anti-EGFR targeting provided by Fab fragments of cetuximab which has been previously investigated in humans [45,80]. A Phase I trial in GBM was completed in 2020 and results are pending (NCT03603379). To our knowledge, 2X-111 and C225-ILs-Dox are the only targeted liposomes which are currently undergoing clinical trials for glioblastoma.

Transferrin receptor (TfR/CD71) is one of the most frequently targeted receptors in the field of BBB drug delivery, and several antibodies and targeting peptides have been developed, some of which have reached phase I/II trials in humans [81]. Yin and colleagues developed a liposome with a TfR receptor-targeting peptide to improve brain uptake, improving intratumoral drug delivery by approximately 4-fold [61]. Sun and colleagues modified a PEG-PLA polymer 110 nm micelles with TfR-T12 encapsulating paclitaxel [82]. This achieved a ~2-fold increase in brain delivery. In another study, modifying a liposome with aptamers targeting the transferrin receptor resulted in a two-fold increase in brain uptake of the target drug [83]. In another study, liposomes conjugated to a variety of cell penetrating peptides were explored for BBB gene vector delivery. The most effective, targeting the transferrin receptor, improved uptake by the brain by 2.4-fold [84]. Kang and colleagues demonstrated an interesting approach using R17217, a transferrin receptor-specific antibody, and Muscone, a Chinese medicinal ingredient, to modify liposomes encapsulating Doclitaxel. They showed improved BBB penetration and extended survival in a mouse xenograft GBM model, with an approximate two-fold increase in delivery to the brain tumors [85]. In all of these studies, the total amount of drug delivered to the brain was still only a small fraction of the dose delivered to peripheral organs. This may be because there are inherent obstacles to the translation of TfR targeting technologies. For example, TfR is widely expressed in multiple organs, not just the brain, so TfR-based targeting lacks tissue specificity. Mice brain capillaries also express more TfR than their human equivalents, and most TfR which crosses the BBB is recycled and does not remain within the brain [81].

Glioblastoma tumors may be targeted directly by exploiting the inflammatory microenvironment. Since they richly express interleukin 13 (IL-13) receptors, PEGylated doxorubicin-loaded liposomes conjugated to interleukin-13 were developed [43]. These showed excellent results in a mouse model, extending median survival from 25 days to 142 days. IL-4 receptor targeting has also been explored, in combination with focused ultrasound, using a novel peptide (AP-1) selected from phage display [46]. In a mouse model this achieved an improvement in overall survival from 9 days to 13 days without focused ultrasound, and up to 15 days with the addition of ultrasound.

Combining multiple technologies, Zhao and colleagues generated ~100 nm liposomes with an Asn-Gly-Arg (NGR) peptide targeting ligand against CD13, which is highly expressed on glioma cells. The liposome payload contained shRNA, and focused transcranial ultrasound was used to further enhance BBB penetration. They found an approximate 8.5-fold increase in shRNA delivery to rat gliomas and extended survival [56]. However, there are a number of limitations including the stability/longevity of the liposome and the relative weakness of the shRNA payload. The liposome also had a lower transfection efficiency than would be expected of a viral vector.

Targeting the SLC2A1/GLUT1 hexose transporter is also a popular strategy for improving brain uptake of nanomedicines since GLUT1 is highly expressed on BECs and the brain constantly transports glucose and other substrates across the BBB. However, first attempts at “hi-jacking” the GLUT1 transporter only demonstrated marginal improvements in BBB crossing [86]. This may be due to the relatively weak binding of glucose to the transporter, which is unable to pull nanocarriers across the BBB. In a novel approach, Anraku and colleagues prepared 30 nm polymeric micelles from PEG with multiple glucose molecules per nanocarrier, thus allowing stronger binding to multiple GLUT1 transporters [53]. Most importantly, they also determined that manipulating mouse glycemic state was essential, and they observed 20-fold increase in nanocarrier uptake (representing ~6% of the injected dose) upon co-administration of the nanocarriers with glucose after a period of fasting. This is notable because the delivered dose is much higher than typically observed in the field. It is also exciting since many glioma cells highly express GLUT1, which may further improve liposome uptake by tumor tissue.

### 3.3. Clinical Translation of Liposomes

For clinical translation, liposomes face less obstacles than other nanomedicines, since they may encapsulate drugs which are already approved for other cancers, and the lipid components themselves are generally considered safe. There are also several existing liposomal drug formulations on the market and many more in late-stage clinical trials for a variety of applications from antivirals to chemotherapeutics, vaccines and analgesics, which are reviewed elsewhere [63,87]. This illustrates that the barrier to translation is not a problem with liposome technology itself—rather, it is the poor delivery of target molecules across the BBB and overall lack of efficacy.

Most targeting strategies achieve modest increases in drug delivery at best. While targeted liposomes commonly improve passage in in vitro BBB models or slow tumor progression in rodent xenograft models, it is questionable whether those improvements will be clinically significant, especially when the total drug delivered remains a small fraction (typically <less than 2%) of the injected dose. It is also notable from the literature that most GBM xenograft studies can show slower tumor growth and extended survival. However, in fields such as breast cancer, nanomedicine formulations routinely cure tumors in mouse models. Again, this illustrates how formidable an obstacle the BBB is, and that there is considerable room for improvement in our drug delivery technology.

## 4. Inorganic Nanoparticles

Despite considerable research interest in inorganic nanoparticles as therapeutic agents, their clinical use for brain tumor treatment is sparse compared to liposomes. Hyperthermia induced by nanoparticles has also been explored as a method for direct killing of tumor cells. In a 2011 clinical study of 15 nm iron oxide nanoparticle-based thermotherapy, nanoparticles (NanoTherm^®^, MagForce AG, Frankfurt, Germany) were directly injected into tumors of 59 patients, then stimulated to achieve a target temperature of 43 °C. Patient survival was improved compared to historical controls, however the technique still has many limitations, such as necessitating removal of all dental implants, and some seizures following treatment [44]. This treatment was approved in the European Union in 2011 and has seen limited use in four clinics in Europe, treating around 100 patients in total [88]. To our knowledge, this is the only metal nanoparticle-based treatment for GBM which is approved by a regulatory agency.

Other inorganic nanoparticles such as silica, gold, iron-based IONPs and carbon-based nanoparticles for BBB theranostics are recently reviewed elsewhere [41,89].

## 5. Biomimetic Approaches to BBB Drug Delivery

Previously, we have described liposomes as a mechanism of BBB drug delivery. However, there is concern about peripheral toxicity, neurotoxicity, or other adverse reactions. This is especially true for nanocarriers for those using heavy metals or non-degradable polymers [31]. Biomimetic approaches seek to use, or exploit, naturally occurring mechanisms of BBB passage which would, in theory, carry less risks and have greater efficacy. The simplest form of biomimicry may be conjugation of BBB carrier ligands such as glucose or transferrin to cloak nanocarriers, as described previously. Next, we describe advanced biomimetic approaches.

### 5.1. Cells as Trojan Horse Carriers of Nanomedicines

Immune cells such as macrophages can cross the BBB and navigate into damaged tissues [90]. Therefore, by targeting those cells for nanomedicine uptake in the periphery, they may then act as carriers or shuttles to carry drugs across the BBB to the desired target area. This “trojan horse” approach has been used as early as 2008 when De Palma and colleagues used monocytes to delivery IFN-alpha to brain tumors [91]. Macrophages are able to cross the vascular wall, co-delivering nano-carriers into the extravascular space, including to brain tumors [92,93]. This approach has been used by our group to design liposomes which use aptamer targeting to attach to monocytes and are then brought to tumor sites [62].

Mesenchymal stem cells (MSCs) and neural stem cells (NSCs) also have the ability to cross the BBB and can home into tumors, presumably due to chemoattraction from cytokines in the tumor microenvironment [15]. This gives rise to a number of exciting possibilities such as using MSCs as delivery vehicles for chemotherapeutic agents, or modifying the cells themselves to secrete therapeutic compounds such as anti-angiogenic cytokines or BBB permeability enhancers [94]. This was demonstrated in principle by Bago and colleagues who genetically engineered NSCs to secrete tumoricidal gene products and injected them intracranially [95]. Due to their inherent tumor-seeking properties, NSCs were able to migrate from the contralateral brain to the tumor side and induce apoptosis of human U87 GBM cells. Unfortunately, the researchers did not explore whether these cells could achieve the same results following intravenous injection. MSCs and NSCs have been safely used in hundreds of human clinical trials for many indications, which is encouraging for the possibility of clinical translation. However, there are barriers to translation of exogenous stem cell therapies, including the cell source, cell preparation, cell fate following injection, and uncertain criteria for quality/efficacy determination prior to injection. To this end, if nanomedicines could target existing endogenous stem cells and use them as BBB-crossing vehicles, this may be a superior strategy.

In another form of biomimicry, cell membranes can be used to coat the surface of nanocarriers as a form of camouflage, thus allowing them to mimic key interactions of the original cell, such as avoidance of immune clearance, tissue specific targeting, and the ability to cross biological barriers [96]. The most common applications in basic research are coating nanocarriers with erythrocyte membranes, stem cell membranes and tumor cell membranes. Cancer cell membrane coatings possess some self-recognition ability, which allows them to then target back to the original tumor [97] Erythrocytes (RBCs) are commonly used owing to their abundance and ease of access, excellent biocompatibility and long circulation time [98]. Erythrocyte-coated nanocarriers may not be ideal for brain targeting, since red blood cells do not normally cross the BBB [99]. However, a RBC membrane-coated, Doxorubicin-loaded PLGA nanoparticle was functionalized with a candoxin-derived peptide which has BBB permeating abilities. In U87 glioma-bearing mice, these nanoparticles achieved a ~3-fold increased delivery to brain tumors, extending mouse lifespan [100]. A microglia membrane-coated nanoparticle carrying Zoledronate achieved a 3-fold increase in brain tumor delivery in a mouse model, by way of CX3CL1/CX3CR1 signaling [101].

Some cancer cells are also able to cross the BBB and form brain metastases, particularly those originating from lung, breast and melanoma tumors. This process is extremely complicated and poorly understood, but likely involves some pre-conditioning of the metastatic niche by secreted products (miRNAs, cytokines, etc.), followed by direct binding of tumor cells to endothelial cells, further ECM remodeling, BBB modulation and migration of tumor cells through the BBB [102,103,104]. By better understanding these mechanisms, they could perhaps be exploited for improving BBB drug delivery in the future, as well as targeted and interrupted to prevent establishment of brain metastasis in the first place. Jia and colleagues performed an interesting experiment using membranes from several cell lines to form fluorescent labelled biomimetic liposomes, including U87 human GBM, C6 rat GBM, B16 melanoma, HepG2 hepatocarcinoma, MCF-7 breast cancer, and bEnd.3 BBB endothelial cells [57]. They found that when incubated with C6 rat glioma cells, uptake of C6 membrane-coated liposomes was 3.9- to 7.9-fold higher, indicating a strong self-recognition. In a rat C6 glioma model, the liposomes showed approximately 2-fold improved uptake. Coupled with photothermal therapy, this reduced tumor volume and extended rat survival. It is possible that the results may be improved upon if exact targeting moieties could be identified and then coated in higher concentrations. For human patients, perhaps it may be possible to extract circulating tumor cell membranes to produce nanocarrier with specific targeting.

As mentioned above, mesenchymal stem cells (MSCs) have natural tumor-homing abilities, through chemoattraction and hypoxia. This technique has been used in basic research for targeting of breast, lung, and other tumors [105]. For example, Zhang and colleagues encapsulated paclitaxel in MSC membrane nanocarriers and demonstrated enhanced tumor drug accumulation and improved clearance of tumors in a mouse model [106]. However, our literature survey indicates that this approach has not been as widely explored in the brain tumor therapy field as it has for other cancers. The feasibility was clearly demonstrated by Hsu and colleagues who coated iron oxide nanoparticles with placenta-derived MSC membranes and tracked their distribution by MRI in a mouse GBM model [107]. Interestingly, they found that nanoparticles coated with membranes from hypoxia-preconditioned MSCs had superior accumulation in the tumor, highlighting that donor origin is highly related to function.

### 5.2. Natural Substrates as Nanocarriers

Earlier, we discussed several studies targeting the transferrin receptor with artificial ligands. Fan and colleagues used ferritin heavy chain (HFn) itself, a natural ligand of the transferrin receptor, as a 12 nm nanocarrier to carry Doxorubicin across the BBB in a mouse model [54]. This approach increased drug delivery to the tumors by approximately 10-fold with only low uptake by healthy brain tissues and no evidence of toxicity, extending mouse survival by almost double.

Albumin, as previously mentioned, is unable to cross the healthy BBB in its blood plasma form. Albumin is a protein approximately 12 × 4 nm, and can be repurposed as a biomimetic nano-carrier which can carry chemotherapeutic drugs across the BBB [108,109]. Abraxane^®^, approved by the US FDA in 2005, is a paclitaxel-albumin nanoparticle which is used clinically for breast cancer treatment, demonstrating the safety of this platform. A new ~100 nm albumin nanoparticle formulation of rapamycin, termed ABI-009, has shown the ability to accumulate in a variety of tumors [58]. ABI-009 is currently under investigation in a Phase II trial of adult patients with glioma, with results expected in December 2022. ABI-009 will be used as a single agent and in combinations with TMZ, bevacizumab, lomustine, radiotherapy or marizomib (NCT03463265). Another ABI-009 trial (NCT02975882) is investigating its safety (Phase I) in pediatric patients in combination with TMZ and irinotecan.

### 5.3. Microorganism-inspired Nanomedicines

Some pathogenic microorganisms can cross the intact BBB and infect the brain, either by paracellular transport or by charge and ligand-mediated transcytosis [110]. Therefore, there it is possible to design nanomedicines to mimic these mechanisms. Common receptors in pathogen transcytosis-based brain entry are the transferrin receptor, insulin receptor and LRP1/LRP2. *Treponema pallidum*, the causative agent of syphilis, interacts with platelets, allowing it to cross the BBB as well as the placental and retinal barriers [111]. Using an approach inspired by *Cryptococcus neoformans*, a cause of fungal meningitis, Aaron and Gelli used a secreted fungal metalloprotease, Mpr1, as a means of increasing quantum dot penetration through an in vitro BBB model [112]. Although this work is preliminary and was not tested in vivo, the approach of harnessing microbial brain invasion strategies is certainly worth further exploration.

Vesicles derived from mutant *Salmonella typhimurium* have been used as nano-carrier (~400 nm) drug delivery vehicles [113]. Termed “minicells”, these vesicles can be loaded with drugs or shRNA/siRNA and be targeted using bi-specific antibodies and have shown the ability to cross the BBB [114]. In 2015 this technology underwent an open-label Phase I/II trial in 14 GBM patients using doxorubicin-loaded minicells with surface anti-EGFR targeting antibodies [50]. Unfortunately, the trial was unable to detect any improvements in progression-free survival or overall survival, though the treatment demonstrated overall acceptable safety. Side effects included fever and cytokine elevation, even though patients were given IV dexamethasone and promethazine, and oral paracetamol. This is most likely due to the bacterial origin of the minicells which express lipopolysaccharide, which is certainly a limitation of this approach. This product is now commercialized as the EnGeneIC DreamVector (EDV^TM^). In 2018, pharmacokinetic studies in large animal models were published showing improved doxorubicin delivery and extended survival in a mouse neuroblastoma model [115].

Use of viral vectors such as adeno-associated virus serotype 9 (AAV9) is an attractive approach for brain tumor gene therapy. Modified AAV9 (AAV-PHP.B and AAV-PHP.eB) have shown preferential targeting of the CNS in animal models [116]. Use of AAV-based gene therapy approaches for GBM have been recently reviewed elsewhere [117].

## 6. Extracellular Vesicles and Exosomes

### 6.1. Introduction to Extracellular Vesicles

**Figure 2:**
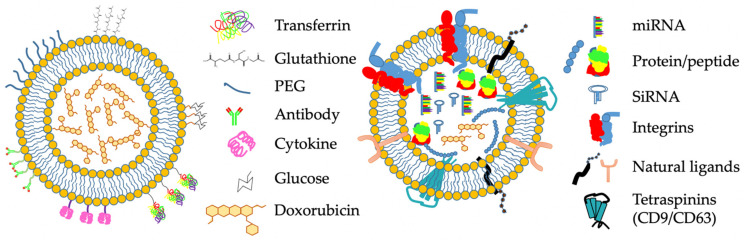
Liposomes and exosomes as BBB-permeating drug nanocarriers. **Left**: Schematic diagram of a liposome. In the simplest form, a liposome comprises a lipid bilayer membrane and a drug payload (e.g., Doxorubicin, shown here). Liposomes are commonly modified with poly(ethylene glycol) (PEG) to improve circulatory properties. As means of improving BBB specificity, substrates such as cytokines, glucose, transferrin or glutathione can be conjugated onto the liposome surface. Antibodies against specific targets may be added. **Right**: Schematic diagram of an exosome. Exosomes are membrane-bound vesicles approximately 100 nm diameter. Their surface is decorated with a variety of natural ligands and receptors, which may have targeting properties. Exosomes contain naturally occurring payloads of proteins, peptides and nucleic acids (miRNA, siRNA etc.). They may also be loaded with exogenous compounds, such as Doxorubicin. Their overall complexity is far greater than liposomes.

Extracellular vesicles (EVs) are naturally occurring, nanoscale membrane-bound vesicles secreted by cells. The term “extracellular vesicles” encompasses microvesicles (100–1000 nm), exosomes (30–150 nm) and apoptotic bodies (50–5000 nm) which each vary based on their content, function and biogenesis [118]. Human body fluids are rich in EVs which serve as a means of communication between distant tissues [119]. EVs in the blood may also originate from platelets and the bacterial microbiome [120]. Since exosomes are formed by budding off from cell membranes, they have highly complex compositions, varying by lipid and cholesterol makeup, protein content and protein modifications. Therefore, exosomes have the inherent ability to bind and interact with specific target cell receptors, which is an advantage over most synthetic nanomedicines [121]. A schematic showing exosome structure and a comparison to liposome structure is shown in Figure 2.

Of all EVs, exosomes have attracted the most research attention due to their small size. They may carry complex biological cargos of lipids, nucleic acids including micro RNAs (miRNAs), and peptides including growth factors, cytokines, heat shock proteins and enzymes [118]. Interestingly, EV contents do not necessarily have the same composition of the originating cell, and protein contents may vary by as much as 100-fold compared to the original cell [118].

Exosomes can be used as standalone therapies or combined/loaded with other therapeutics. As standalones, exosomes do not have notable intrinsic tumor-killing abilities and so are most useful for anti-inflammatory or immune-modulatory purposes. For CNS applications, this lends itself towards neurodegeneration or traumatic brain injury where modulating inflammation can be beneficial. However, exosomes can be modified into tumor-killing agents, via modification of the parent cell, such as overexpression of miRNAs which inhibit cell growth or inhibit angiogenesis [122]. Alternatively, exosomes can be loaded with exogenous products and thus can serve as drug and gene delivery vehicles. For example, exosomes were engineered to express shRNA and siRNA targeting oncogenic KRAS and were successful at preventing metastasis and extending survival in several mouse models of pancreatic cancer [123].

### 6.2. EVs as Vehicles for Brain Tumor Delivery

For delivery to brain tumors, exosomes are a promising option which are, as of mid-2021, still much less explored than liposomes, nanoparticles and other nanotechnologies. A recent study found that HEK293T-derived exosomes crossed an in vitro BBB model in greater quantities than liposomes or polymeric nanoparticles [124]. They also displayed superior therapeutic efficacy in a mouse model. An elegant study from Morad and colleagues showed that exosomes derived from brain-seeking breast cancer cells can breach the BBB by transcytosis, aided by decreasing endothelial rab7 expression [125]. Interestingly, blood-derived EVs have also been found to express transferrin-TfR complexes on their surface, which aids their uptake into the brain [126]. However, there is variance in precisely which cells produce exosomes capable of crossing the BBB. Studies have shown that exosomes from endothelial cells interact with the BBB, whereas exosomes from cultured GBM cells could not. However, hypoxic GBM cells produced exosomes capable of modulating BBB permeability [127]. Cancer cells also produce exosomes which weaken the BBB, which may be a way to pre-condition the brain for metastasis [128]. Banks and colleagues compared exosomes from 10 cell lines, including cancerous and non-cancerous lines, and found that although all could cross the BBB in some quantity, the extent of uptake into the brain varied by more than 10-fold [129]. Exosomes derived from macrophages may be a good candidate since they were able to cross the BBB and delivery brain-derived neurotrophic factor (BDNF) to the brain after intravenous injection [130].

Since some exosomes can cross the BBB, they may be used as delivery vehicles for conventional chemotherapeutic drugs. Drugs may be loaded into exosomes by a variety of methods including simple incubation and passive uptake, sonication, electroporation, freeze–thaw or use of surfactants [127]. A study by Yang and colleagues showed that bEND.3-derived exosomes could be loaded with doxorubicin and paclitaxel (7.3 ng and 132.9 ng of drug per 1 µg exosome) and showed better therapeutic efficacy than free drug in a zebrafish tumor model [131]. Zhuang et al. loaded curcumin and a Stat3 inhibitor molecule into exosomes derived from EL-4 cells and administered them intranasally in a mouse brain tumor model [132]. They found that intranasal exosomes were taken up largely by brain microglial cells and reduced inflammatory cytokine expression, thus enabling more apoptosis of tumor cells.

### 6.3. Clinical Translation of Exosomes for Brain Tumor Applications

For clinical translation, exosomes are attractive due to their natural origin, small size, and their low degree of expected toxicity [133]. However, their biological origin and complexity comes at the cost of consistency and reproducibility. Exosomes secreted by different originating cells have diverse effects on target cells. For example, exosomes derived from cultured stem cells have been explored for regenerative medicine, but exosomes derived from metastatic tumors induce changes in healthy cells [134,135]. As described earlier, the efficiency of different cell-derived exosomes in BBB crossing also varies widely and it is unclear what parameters can be used to screen exosomes for likelihood of BBB permeability. Exosome circulatory properties are also not ideal for chemotherapy treatments, since their circulatory half-life is approximately two minutes [122,129].

In addition, there are several challenges which need to be overcome on the road to clinical translation, most of which are practical or methodological issues. Firstly, exosomes have a relatively low yield, particularly if they are being sourced from cultured cells. For treatment of a single human patient, a large volume of defined serum-free culture media is required to avoid contamination by bovine proteins and miRNAs. Secondly, storage of exosomes, even at −80 °C, is known to affect their functional properties and activity [136]. Third, the source of exosomes and their isolation methods (ultracentrifugation, precipitation by polymers, size exclusion chromatography and filtration methods) need to be extremely carefully considered [137]. These methods each influence the properties of isolated exosomes population due to preferential capture and loss of different subpopulations. Recently, size-exclusion chromatography is allowing a more granular approach, whereby individual fractions can be analyzed for their desired effects [138]. Ultracentrifugation is considered the gold standard of exosome isolation, but still does not produce totally pure exosomes (e.g., contamination by plasma lipoprotein particles, protein complexes and viruses), and may damage the exosome membranes [139]. New technologies are also under development including microfluidic isolation methods, nano flow cytometry which can differentiate EV subpopulations.

There also must be means to standardize batches, including definitions of critical quality attributes (e.g., size range, surface markers, protein content, etc.). This is challenging, since exosomes are heterogenous by nature, varying in size, surface markers, cargo, and their effects on recipient cells [140]. Databases such as Vesiclepedia (http://microvesicles.org, accessed on 14 July 2021) and Exocarta (http://www.exocarta.org, accessed on 14 July 2021) aim to allow more precise information sharing about exosome populations and their cargo. In addition to exosomes themselves, the originating cells must also be standardized to ensure the quality of EVs which are produced. For human donors, similar challenges exist since the health condition of the donor will influence the properties of the isolated EVs, including their therapeutic usefulness [139]. For example, exosomes derived from GBM cells showed tumor-promoting transformation of neural stem cells and GBM patients have specific plasma exosomes which reflect their disease condition [141,142]. Therefore, exosomes from GBM patients may not be suitable for autologous therapy. Cell culture conditions including mild cellular hypoxia or stress, media cytokine concentrations and available glucose levels impact exosome protein and nucleic acid contents [135].

Therefore, many quality control experiments may need to be undertaken, including biological assays to confirm the desired function. For example, in brain tumor applications, this may involve confirming that EVs can inhibit vasculogenesis using in vitro assays. The International Society of Extracellular Vesicles (ISEV) is the leading authority who aim to provide best-practice recommendations for EV collection, characterization, reporting and use [143]. However, there is still considerable variability in the research literature.

## 7. Clinical Translation of Nanomedicines for Brain Tumor Treatment

**Figure 3:**
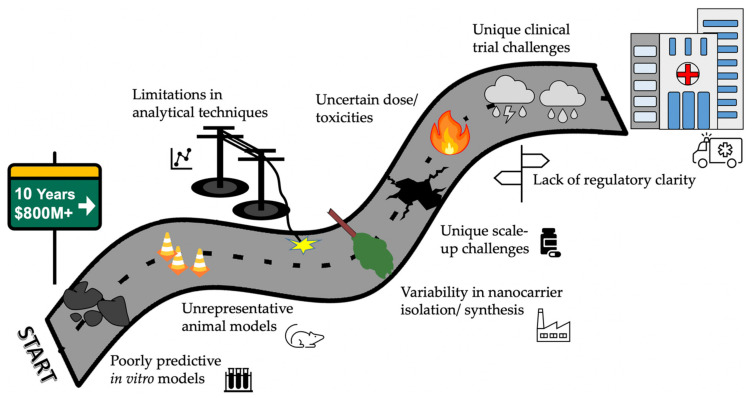
The road to clinical translation of nanomedicines for brain drug delivery contains many obstacles. Scaling up a nanocarrier technology from research/publication-grade to a commercial product in clinical trials is fraught with challenges. Under optimal conditions, it may take five years to reach clinical trials and the best part of a decade to reach full clinical use. Some of the common obstacles are illustrated in the schematic diagram, but this is not an exhaustive list. Many publications rely on poorly predictive in vitro models such as brain endothelial cell monolayers, or unrepresentative animal models such as xenografts. There are serious limitations in measuring nanocarrier distributions and brain uptake, especially in pre-clinical studies. Variability liposome manufacture, or exosomes isolation, also produces heterogenous final products. All pre-clinical products face challenges in scale up from the lab bench, but nanocarriers face many additional hurdles. One of the largest is the regulatory uncertainty, including unclear criteria for determining batch consistency and bioequivalence. Finally, there are unique clinical trial challenges, including the inability to sample brain tissue to quantify drug uptake.

For successful clinical translation, any nanotechnology-based approach needs to be safe and must offer benefits above and beyond the current standard of care. As of mid-2021 there are less than 25 nano-based drugs commercially available for cancer treatment—the vast majority being liposomal formulations of older chemotherapeutic agents such as Doxorubicin, Paclitaxel and Daunorubicin [144]. This is highly disproportionate to the large numbers of early clinical trials and the decades of research funding and publications in the nanomedicine field [145]. In the brain drug delivery field, the ratio is even poorer. A schematic illustrating some of these obstacles is shown in Figure 3.

In the basic research literature, complex approaches such as combining multiple targeting ligands, or combining nanomedicine with other techniques, can successfully increase drug delivery. However, these approaches introduce more variables, which complicates the manufacture and scale-up, and introduces more barriers to regulatory approval. It is likely that the first treatments to reach the market for brain tumors will be simplified approaches using focused ultrasound or liposomal nanocarriers in combination with already-approved drugs such as liposomal doxorubicin or Temozolomide.

Due to the extremely poor prognosis and severe lack of treatments facing GBM patients, regulatory agencies are more permissive than they may be in other indications. For example, Temozolomide was granted US FDA approval based on a small improvement in overall survival from 12.1 to 14.6 months [146]. Bevacizumab was approved for use in GBM patients even though it offers symptomatic relief but no improvement in overall survival [147].

### 7.1. Limitations of In Vitro and Animal Models

In the brain drug delivery area, two large barriers to successful clinical translation are the relative lack of robust pre-clinical models, and the difficulty in conducting human clinical trials. In vitro BBB models typically use human brain endothelial cell lines such as hCMEC D3 or bEND.3, or freshly extracted animal primary BECs, grown on a semi-permeable membrane in a transwell or microfluidic apparatus [8,148]. To improve barrier function, endothelial cells can be co-cultured with supporting cells such as pericytes and astrocytes which increase BEC expression of tight junctions and transporters [149]. These models can demonstrate BBB passage of nanomedicines in an artificial setting. However, expression of targets such as LRP1, TfR, GLUT1, etc., vary depending on the culture conditions, leading to variable outcomes. Furthermore, in vitro models cannot reliably account for safety or efficacy of the formulation inside the human body.

For animal studies of brain tumors, rodents are commonly used; either immunodeficient mice with human cell line xenografts (U-87MG, DBTRG etc.), or immunocompetent animals with rodent GBM cell lines. These models have inherent weaknesses due to their artificial nature, and the differences between animal BBB and human BBB. For example, Syvanen and colleagues investigated brain permeation of three model drugs in rat, guinea pig, minipig, monkey and humans. They found brain:plasma drug concentrations differed by as much as 9-fold, which varied between drugs and between species [150,151]. Nevertheless, rodent models are a useful testing platform due to their ease of access, reliability, and the degree of control available to researchers. Less commonly in basic research, patient-derived tumor xenografts are used. Another limitation is the analytical techniques used for studying BBB penetration. Typically, the animal is sacrificed, the circulatory system is perfused to remove drug from brain capillaries, brain/tumor tissue is removed, homogenized and the drug concentration is measured. Unfortunately, these techniques are invasive and can provide only one sample per animal, thus requiring large numbers of animals for detailed dose/time/response studies. Furthermore, they are indirect measurements, since homogenizing a piece of brain tissue does not reliably indicate how much active drug was released and subsequently taken up by tumor cells or how much was present in the encapsulated form. In a study by Dai and colleagues, 0.7% of injected nanoparticles were present in the solid tumor mass of CD-1 nude mice [152]. However, upon detailed inspection, only 0.0014% were delivered to the tumor cells; the rest were either trapped in extracellular spaces or taken up by macrophages. New techniques such as microdialysis and cerebral open flow microperfusion could be used to continuously measure drug concentrations in brain interstitial fluid [153]. However, these are technically challenging to implement in most research labs. In an ideal world, it would be possible to confirm/quantify drug delivery using standard clinical imaging technologies such as MRI, X-ray, PET, SPECT or CT.

### 7.2. Limitations in Technical and Analytical Methods

In human trials, sampling the cerebrospinal fluid (CSF) is often used as a proxy measurement for brain drug delivery [154]. Although CSF concentration shows nanomedicine entry to the CNS, it does not reliably confirm drug delivery to a target site such as a tumor [155]. The optimal approach is to give an experimental treatment prior to scheduled surgery tumor resection surgery so that the tumor drug concentration can be directly measured [24]. However, this would be limited to clinical trials and is not practical for long courses of treatment.

In addition, there are limitations to the common characterization methods used in the field. Transmission electron microscopy (TEM) relies on drying of the sample which does not represent their condition in the body. Dynamic light scattering (DLS) estimates particle size based on an assumption of spherical shape. Consideration must be given to the protein corona which is established rapidly after any non-biological material is introduced to the blood and evolves in a dynamic manner depending on protein affinity and abundance. Adsorption of plasma proteins, including anti-PEG antibodies, alters the hydrodynamic diameter, charge and biological recognition of the material, which may therefore affect its pharmacokinetic and pharmacodynamic properties [156].

Furthermore, encapsulation efficiency, and thus the ratio of active molecule to carrier, varies widely depending on the drug and lipid combination, as does the drug release rate in circulation. Drug release is typically measured by dialysis of the free molecule through a membrane at a set temperature and pH. However, this does not necessarily represent the drug release rate in the bloodstream. For example, anti-PEG antibodies may cause premature drug release [66]. Recently, there have been calls for more standardization of characterization methods in the nanomedicine field [157].

### 7.3. Dosing and Route of Administration

The pharmacology of nano drugs is highly complex, since the active molecule can simultaneously exist in the nanomedicine-bound state in circulation, as a free drug in circulation, and as a drug bound to carrier proteins. Uptake by the brain may also be in the form of an encapsulated drug, bound drug or free drug, and the amount of efflux by the BBB also determines the drug effectiveness. Generally, only the unbound drug present inside the brain parenchyma would be considered as therapeutically relevant. The unbound brain-to-plasma exposure ratio (K_p,uu,brain_) can be used to evaluate brain uptake and make predictions about peripheral toxicity [109]. Since a successful nanomedicine would display greater BBB permeability than its naked counterpart, toxicity to brain tissue itself would also increase [158]. This is particularly true for chemotherapeutic drugs where acute and chronic neurotoxic effects may occur. Since GBM affects both pediatric and elderly age groups, there is a need to confirm the appropriate dosage for each patient population since there are known differences in biodistribution of polar and non-polar drugs with age [159]. In addition, the effects of renal or liver impairment on pharmacokinetics of nanomedicines is less researched than for free drugs. Most nanomedicine formulations are given by intravenous administration in animal models. However, oral dosing (such as Temozolomide) is preferred by patients. Intranasal delivery, mentioned previously, also allows brain tumor drug delivery in a minimally invasive administration [160].

### 7.4. Nanotoxicity Symptoms, Monitoring and Prevention

The term “nanotoxicity” describes the side effect of nanomaterials on human health and has been mostly studied in animal models. Adverse effects of nanoparticles can occur in many organs and often originate from disruption of cellular redox and subsequent ROS activation, or by disruption of cell membranes [31,109]. However, criteria for defining and measuring nanotoxicity are undefined, and great variation exists in the literature which relies on in vitro viability assays, ROS assays or electron microscopy of cell membranes—none of which are amenable to clinical measurement of nanotoxicity. For targeting of brain disorders this is critically important since disturbances to neuronal membrane integrity may result in erroneous action potentials, cell death, edema, and other consequences. In most basic research, there is no attempt to quantify remaining impurities such as residual organic solvents. Normally, the final product testing follows the National Pharmacopeia of each country in which the criteria and test method is described in detail for each active compound in a specific dosage form. However, very few nanomaterials monographs are found in European and American Pharmacopeias (Ph. Eur. and USP) [161]. Therefore, to conduct the translation process for nanomedicine, the development of specific criteria and analytical method for nanotechnology-based products is necessary.

### 7.5. Nanomedicine Regulation

Manufacturing and production of any commercial drug needs to be reproducible and scalable, which can be especially challenging for nanomedicine formulations. Nanomedicines are far more complex than small molecules, and the regulatory framework is also much less clear and varies between agencies. For example, The European Commission defines nanomaterials as those formed from individual particles between 1 and 100 nm (2011/696/EU), although it is clear from the literature that many nanomedicines are larger than 100 nm. The European Medical Agency (EMA) formed the European Nanomedicine Expert Group in 2009, and the EMA has since established guidelines (not regulations) for nanomedicine preparation [162]. There is also a European Nanomedicine Characterization Laboratory (EU-NCL) and a US NCL which “aims to provide expertise in physical, chemical, in vitro and in vivo biological characterization of nanoparticles for medical use.” [163] The UK Medicines and Healthcare Products Regulatory Agency (MHRA) treats new nanomedicine applications on a case-by-case basis, as does the US FDA [164]. Recently, the concept of “nanosimilar” has emerged to replace the “sameness” requirement for follow-on drugs [165]. Recently, a “REFINE” project has been established to set up the criteria for regulating nanomedicine in clinical use [166].

Due to a lack of a formal regulatory framework, there are no specific definitions for which critical nanomedicine physiochemical properties (size, charge, ligand density, polydispersity, etc.) must be characterized and reported [167]. Thus, establishing batch-batch consistency or bioequivalence is challenging, since these properties largely dictate the behavior of nanomedicines in the body. In many instances it is unclear which aspect of a nanomedicine defines the “active ingredient”, since the drug molecule, carrier and surface targeting molecules are all critical to the function of the final product [168]. To this end, the “case-by-case” approach is pragmatic and more flexible, where the potential benefits and risks of each individual product can be assessed.

## 8. Conclusions

Brain drug delivery is a serious challenge, illustrated by decades of scientific research which has borne little fruit. Survival rates for GBM remain stubbornly low despite great improvements for most other cancers. Nanomedicines have great potential to improve therapeutic outcomes for brain tumor patients. The field is exciting and developing quickly, with research groups around the world creating and discovering new liposomes, nanoparticles, extracellular vesicles and medical devices for improving brain drug delivery. However, there are still significant challenges, particularly in creating solutions which are highly efficacious, clinically feasible and realistic. Most published research shows technologies which produce moderate, but not transformative, improvements in brain drug delivery. As a result, most of the promise of these technologies has not yet been realized in the clinic. There are also significant obstacles with the translation of nanomedicines to the clinic, including lack of regulatory clarity. Pre-clinical evidence needs to be robust, using several methods to fully characterize nanomedicines, understand the properties essential for function, and to demonstrate their efficacy in multiple in vitro and in vivo models. Nevertheless, we are positive about the future direction of the field and believe that the great promise will ultimately be realized.

## Figures and Tables

**Table 1:** Key Studies in the Field of Nanocarrier-based Brain Tumor Treatment.

Year	Nanocarrier	Category	Stage	Species	Key Findings	References
2009	PEGylated Liposomal Doxorubicin	Untargeted liposome	Phase II	Human GBM	No benefit in GBM patient compared to TMZ alone	[42]
2009	IL-13-conjugated liposomal Doxorubicin	Targeted liposome	Basic research	Mouse U87 xenograft	Dramatic increase in OS from 25 d to 142 d	[43]
2011	Iron oxide nanoparticle	Inorganic nanoparticle	Phase I	Human GBM	Single-arm study. OS 13.4 months. Numerous adverse events noted	[44]
2012	Anti-EGFR immunoliposomal Doxorubicin	Targeted liposome	Phase I	Human (Multiple tumors)	Acceptable safety and tolerability. Recommendation for Phase II	[45]
2012	AP-1-conjugated liposomal Doxorubicin targeting GBM IL-4R	Targeted liposome	Basic research	Mouse 8401 xenograft	Combined with FUS. ~5-fold increase in Dox delivery. Improved MS by 67%.	[46]
2014	Doxorubicin-loaded Liposome	Targeted liposome	Basic research	Healthy mice	GSH-targeted increase BBB permeability	[47]
2014	Glutathione PEGylated liposomal Doxorubicin (23B-101)	Targeted liposome	Basic research	Healthy rat	4.8-fold increase in brain-to-blood ratio	[48]
2014	Glutathione PEGylated liposomal Doxorubicin (23B-101)	Targeted liposome	Phase I	Human GBM and brain metastasis	Good safety profile and preliminary efficacy	[49]
2015	Doxorubicin-loaded bacteria-derived minicell targeting EGFR	Targeted minicell	Phase I	Human GBM and brain metastasis	Median OS 9 months. Cytokine elevations	[50]
2015	Doxorubicin-loaded liposome following focused ultrasound	BBB pre-weakening	Basic research	Healthy rat	Dox reached therapeutic concentrations. Histological changes at target site.	[51]
2017	Iritinocan-loaded Liposome	Untargeted liposome	Phase I	Human GBM	No unexpected toxicities. Follow-up to explore CED ongoing (NCT02022644)	[52]
2017	PEG-based nanocarrier targeting GLUT-1 (no active drug payload)	Targeted liposome	Basic research	Healthy mouse	20-fold increase in uptake, linked to glycemic status of animals	[53]
2018	Doxorubicin Ferritin heavy chain (HFn)	Natural nanocarrier	Basic research	Mouse U87 xenograft	Extended MS from 16 d to 30 d	[54]
2018	Dox-loaded thermosensitive liposomes stimulated by intracranial probe	Thermosensitive liposome	Pre- clinical	Healthy dogs	Dox concentration increased from 0.11 to 0.74 µg/g. Histological evidence of damage to brain	[55]
2018	shRNA-loaded liposome	Targeted liposome	Basic research	Rat C6 GBM	8.5-fold increased drug delivery. Extended survival time	[56]
2019	Glioma cell membrane-coated liposome with photosensitivity	Targeted liposome	Basic research	Mouse C6 GBM	Increased liposome delivery, increased survival. Allowed labeling of tumor margins	[57]
2019	Rapamycin-albumin nanoparticle (ABI-009)	Natural nanocarrier	Phase I	Human (Sarcoma)	PR+SD in 93% of patients. Reasonable safety profile. Not yet applied to GBM	[58]
2019	Liposomal Doxorubicin or Temozolomide delivery via FUS	BBB pre-weakening	Phase I	Human GBM	First in human. 7-fold increase uptake of drug in one patient	[24]
2019	Liposomal Doxorubicin combined with BBB pre-weakening	Untargeted liposome	Pre- clinical	Mouse U87 xenograft/ Minipig	6.4-fold increase in healthy mice. 13.6-fold increase in GBM mice. ~3-fold in healthy pigs	[59]
2020	Liposomal Irinotecan	Untargeted liposome	Phase I	Human (brain metastasis)	Notable anti-tumor effects in heavily pre-treated breast cancer brain metastasis patient	[60]
2020	Transferrin-receptor-targeted-peptide liposome	Targeted liposome	Basic research	Mouse H1975 xenograft	A 4-fold increase in drug delivery. Extended MS from 15 to 33 days	[61]
2021	NP with aptamer-based monocyte targeting	Cell-based ‘trojan horse’	Basic research	Mouse KPC	Gemcitabine-bearing NPs significantly increased survival in metastatic tumors	[62]

MS, median survival; OS, overall survival; GBM, glioblastoma multiforme; PR, partial response; CR, complete response; SD, stable disease; CED, convection enhanced delivery; FUS, focused ultrasound; NP, nanoparticle; Dox, doxorubicin.
